# Assessment of Racial Misclassification Among American Indian and Alaska Native Identity in Cancer Surveillance Data in the United States and Considerations for Oral Health: A Systematic Review

**DOI:** 10.1089/heq.2023.0252

**Published:** 2024-06-26

**Authors:** Amanda J. Llaneza, Alex Holt, Julie Seward, Jamie Piatt, Janis E. Campbell

**Affiliations:** ^1^ Southern Plains Tribal Health Board, Oklahoma City, Oklahoma, USA.; ^2^ Department of Biostatistics & Epidemiology, Hudson College of Public Health, University of Oklahoma Health Sciences Center, Oklahoma City, Oklahoma, USA.

**Keywords:** racial misclassification, American Indian/Alaska Native, cancer surveillance, oral health, health equity

## Abstract

**Introduction:**

Misclassification of American Indian and Alaska Native (AI/AN) peoples exists across various databases in research and clinical practice. Oral health is associated with cancer incidence and survival; however, misclassification adds another layer of complexity to understanding the impact of poor oral health. The objective of this literature review was to systematically evaluate and analyze publications focused on racial misclassification of AI/AN racial identities among cancer surveillance data.

**Methods:**

The PRISMA Statement and the CONSIDER Statement were used for this systematic literature review. Studies involving the racial misclassification of AI/AN identity among cancer surveillance data were screened for eligibility. Data were analyzed in terms of the discussion of racial misclassification, methods to reduce this error, and the reporting of research involving Indigenous peoples.

**Results:**

A total of 66 articles were included with publication years ranging from 1972 to 2022. A total of 55 (83%) of the 66 articles discussed racial misclassification. The most common method of addressing racial misclassification among these articles was linkage with the Indian Health Service or tribal clinic records (45 articles or 82%). The average number of CONSIDER checklist domains was three, with a range of zero to eight domains included. The domain most often identified was Prioritization (60), followed by Governance (47), Methodologies (31), Dissemination (27), Relationships (22), Participation (9), Capacity (9), and Analysis and Findings (8).

**Conclusion:**

To ensure equitable representation of AI/AN communities, and thwart further oppression of minorities, specifically AI/AN peoples, is through accurate data collection and reporting processes.

## Introduction

American Indians and Alaska Natives (AI/AN) comprise about 2.9% of the total U.S. population.^1^ However, in parts of the United States, some counties are well over 50% of the population.^2^ Those AIs who are members of a state or federally recognized tribe have health coverage through the Indian Health Services (IHS), Tribal Health Services, or in Urban Indian centers as per U.S. treaty responsibilities. Misclassification of race is a significant problem when describing health disparities and equity. With many decisions, including appropriate funding, organizational practices, and quality measures, accurate data are paramount. In short, this has been problematic for AI/AN populations as they are often racially misclassified in datasets, which are critical for decision-making. Although the purpose of this article is centered on cancer disparities and racial misclassification in cancer registries and databases, it is important to note that racial misclassification can be found in other major databases used to monitor health trends. For example, oral health data rely heavily on publicly available databases and, depending on how race data are collected, may be subject to varying amounts of racial misclassification.^3^ If the data are not linked with IHS patient files, this misclassification in oral health data may skew or alter actual trends in the data.

In the United States, the age-adjusted cancer mortality rate (AADR) was 146.6/100,000, which was the second leading cause of death in 2021 following only heart disease (AADR 173.8/100,000) for both the overall population and the AI/AN population.^4^ It is evident that pervasive and long-standing health disparities exist within the AI/AN communities. Some areas across the United States (such as the Northern and Southern Plains) experience significant health disparities among AI/AN males for colorectal, liver, and stomach cancer incidence. Among AI/AN women this includes liver, stomach, kidney and renal cell, cervix, and gallbladder cancer incidence.^5–7^ Thus, despite their small numbers compared with the general U.S. population (an estimated 5.9 million in 2020^1^), the AI/AN population is important in eliminating cancer incidence disparities.

There is clear evidence that oral health is critical for disease incidence and survival, including all-cause mortality^8–14^ but specifically heart disease,^14–18^ dementia,^19^ stroke,^17^ and cancer.^15–18,20–24^ Cancers associated with either tooth loss or periodontal disease include oral cancer,^25–27^ esophageal cancer,^23,26^ stomach cancer,^23,25,26^ colorectal,^28^ liver cancer,^23^ and lung cancer.^22,29^ These studies primarily focus on periodontal disease or tooth loss as a measure of oral health and there are often inconsistencies in the assessment of periodontal disease and tooth loss (whether self-reported or measured).^21^ In 2020, AI/AN populations had the highest proportion of any racial or ethnic group in the United States (45.2%) not having seen a dentist in the past year and the most likely (23.7%) to be age 65 or older and have all of their teeth removed.^30^

Research has shown that misclassification of AI/AN peoples exists across various databases in research and clinical practice.^31,32^ The implications are far-reaching as it can distort reported population estimates of outcomes not only of the AI/AN population but other races as well. This is an issue of numerator and denominator dissonance, and the direction of the bias is underestimation of risk for AI/AN populations. Currently, cancer incidence data often include patients who are linked to the IHS master patient file. The process of data linkage is that state vital statistics and/or cancer registry records are linked with the IHS patient registration database.^33^ This identifies AI/AN records previously misclassified as non-native in the state or cancer data, thus improving data quality and reducing racial misclassification.^33^ Efforts have been made by major databases such as the National Cancer Data Base and hospital cancer registries to link historical cancer data to IHS patient files, but this work is not complete. To date, the only regularly linked data are cancer registries, the Surveillance, Epidemiology, and End Results (SEER) Program, and the National Program of Cancer Registries (NPCR), which released the aforementioned linked data. Thus, cancer incidence and mortality data are linked. Linkage of IHS data and the National Death Index mortality data for other diseases has been done as well.^34^ The objective of this literature review was to systematically evaluate and analyze publications focused on racial misclassification of AI/AN racial identities among cancer surveillance data. Specifically for the purposes of this article, the authors reviewed cancer disparities because of the ongoing data linkages between cancer registries and the IHS through the Centers for Disease Control and Prevention (CDC).

## Methods

The Southern Plains Tribal Health Board (SPTHB), a nonprofit tribal public health organization dedicated to serving the 43 tribal nations throughout Oklahoma, Kansas, and Texas, conducted this systematic literature review. The authors checked PROSPERO in July 2023 but were unable to register the article because data extraction was already completed. As of July 2023, there were no relevant protocols published. The authors used the 2020 PRISMA (Preferred Reporting Items for Systematic Reviews and Meta-Analyses) statement for this review.^35^ To standardize data abstraction and determine consensus, COVIDENCE, the systematic review management system was used. Article screening, data extraction, and assessments were done independently by two reviewers (A.J.L. and A.H.) to reduce errors and to detect any differences in interpretation between extractors. Any inconsistencies were discussed and resolved by a third reviewer (J.E.C.). For all included studies, the following were extracted: bibliographic information, study design, exposure(s), and outcomes, including definitions, characteristics of study participants, numerical results, and sample size. Specifically, cohort year, region/state, name of registry/data source, cancer type evaluated, if misclassification was discussed (yes/no), how misclassification was accounted for, and race/ethnicity data collected were abstracted.

The search strategy was designed to access published articles. Terms identified and their respective synonyms were used by corresponding databases and were used in an extensive search of the literature. Full copies of articles identified by the search, and considered to meet the inclusion criteria, based on their title, abstract, and subject descriptors, were obtained for data synthesis. Sources of published articles included Ovid MEDLINE, PubMed, Web of Science, Scopus, and University of New Mexico Native Health Database with the searches taking place from November to December 2022. The search terms used included (*American Indian* OR *Native American* OR *Alaska Native* or *AIAN*) AND (*misclass or mis-class or miscategory* or *mis-category or miscode or mis-code*) AND (*cancer* or *malignant*). After assessment of eligibility, the articles were qualitatively assessed. The importance of the results in terms of clinical and public health relevance was discussed.

The goal of the search strategy was to identify any type of cancer to better understand racial misclassification among AI/AN populations. However, the authors also wanted to specifically highlight any studies that pertain to oral and pharyngeal cancer in the Results section to introduce translational applications of findings to oral health.

### CONSIDER Statement

There are minimal guidelines related to reporting of research that involves Indigenous peoples.^36^ The CONSolIDated critERia (CONSIDER) for strengthening the reporting of health research involving Indigenous peoples statement was created to provide a checklist for the reporting of health research involving Indigenous peoples.^36^ It establishes 8 research domains and 17 criteria for the reporting of equitable research practices with Indigenous peoples.^36^ The CONSIDER checklist domains were used during data extraction for this systematic literature review.

## Eligibility Criteria

This review considered all English-language studies that involve human subjects on the racial misclassification of AI/AN identity among cancer surveillance data. Exposures of interest considered were AI/AN communities in the United States. The primary outcomes of interest were reporting racial misclassification among cancer surveillance data. The review considered observational studies, including cohort studies, retrospective chart reviews of large centers, and cross-sectional studies.

**FIG. 1.**
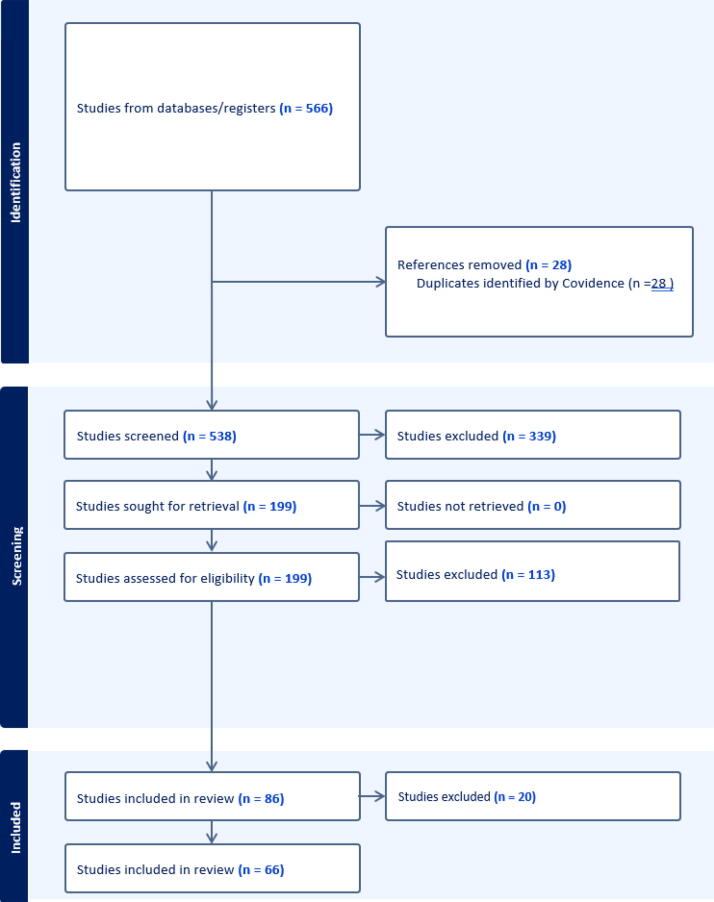
Study selection flow diagram, *n* = 66.

A total of 566 articles were selected from the various databases listed above (Fig. 1). Through COVIDENCE, 28 articles were removed as duplicates. A total of 538 articles were screened through title and abstract review, where 339 studies were irrelevant based on the eligibility criteria. A total of 199 were screened with full-text analysis to assess eligibility and 113 were excluded. A total of 86 articles were assessed for full-text data abstraction, of which 20 were excluded. A total of 66 articles were included for the analysis.

## Results

A total of 66 articles were analyzed for this review according to the PRISMA statement and the CONSIDER statement.^35,36^ The published year for the articles ranged from 1972 to 2022.^5–7,33,37–98^ Cohort years for the data varied, with the earliest cohort year being 1950–1967^46^ to the latest being 2014–2018.^7^

Of the 66 articles on cancer surveillance among populations in the United States, 12 (18%) did not discuss racial misclassification or did not account for misclassification in their study.

### Articles That Discussed Misclassification

A total of 55 (83%) of the 66 articles discussed racial misclassification (Table 1). The dates of publication for these articles ranged from 1986 to 2022. Of these articles, the oldest and largest cohort of data, or range of data collected, was from 1969 to 1994.^54,55^ The most recent cohort of data that addressed misclassification was from 2014 to 2018.^7^ The most common cohort of data (17 articles or 31%) used was from 1999 to 2004.^6,40,42,51,58,62,65,75,80,84,91–94,97^ There were 15 articles (27%) that used cohorts of data that bridged the gap between 2004 and 2018.^43,44,49,56,57,59–61,66,68–71,75,76,83,90^

**Table 1.** Table of Results, *n* = 66

Authors	Year	Name of registry or data source	Misclassification data discussed	How misclassification data accounted for (if applicable)
Anderson RN, et al.	2003	Populations from the 2000 census bridged to single race, death certificates filed in the 50 states and DC; Vital Statistics Cooperative Program	No	N/A
Baquet CR	1996	American Samoan data from Hawaii SEER, AN data from Lanier and Knutson 15-year summary of AN cancer data, AI from NM SEER	No	Methods mention New Mexico SEER as “relatively free of misclassification errors”
Becker TM, et al.	2002	Cancer registries of Idaho, Oregon, and Washington	Yes	Accounted for using linkage analysis (52.2%) originally misclassified by state registries as race other than AI/AN with NW Tribal Registry
Becker TM, et al.	2008	NPCR and SEER/NCI; IHS enrollment	Yes	All case records from each state were linked with the IHS patient registration database to identify AI/AN cases misclassified as another race
Bleed DM, et al.	1992	Montana Central Tumor Registry; IHS databases	Yes	IHS linkage
Bliss A, et al.	2008	NPCR and SEER/NCI and IHS enrollment records	Yes	States worked with the IHS to link cancer registry databases with IHS patient registration data. This linkage effectively identified those individuals who may not have been classified as AI/AN in the state cancer registries but were known to be members of federally recognized tribes and enrolled in the IHS health care system.
Bruegl AS, et al.	2020	Population-based cancer data from the cancer data registry of Idaho, Oregon, and Washington	Yes	State cancer registry data were corrected for AI/AN racial misclassification through probabilistic linkages with the Northwest Tribal Registry—a database of personal identifiers for AI/AN patients seen in Indian Health Service, tribal, and urban Indian health clinics in Idaho, Oregon, and Washington. State death certificates were corrected for AI/AN racial misclassification using similar methods as those used for the incidence data.
Campbell JE, et al.	2014	Oklahoma Central Cancer Registry; Cherokee Nation Cancer Registry, IHS files	Yes	To identify AI/AN cancer cases that were misclassified as other races, OCCR works with IHS to link cancer registry records with IHS patient registration file
Cobb N, et al.	1998	Death certificates and U.S. Census data	Yes	The study sample included AI/ANs who live in the service-area counties of the IHS, where the concentration of Native Americans is high
Creagan ET, et al.	1972	Death certificates provided by the NCHS to the NCI	No	N/A
Creswell PD, et al.	2013	Six tribal and/or urban Indian clinics records; Wisconsin cancer reporting system	Yes	Compared the results with the state cancer registry to evaluate missing or racially misclassified cases; data linked with Wisconsin registry; 10/13 clinics participated
Davidson AM, et al.	1996	Public and private medical facilities in the state of Alaska; Alaska SEER; Cancer Surveillance System Fred Hutchinson Cancer Research Center	No	N/A (data came directly from IHS clinics)
Dougherty TM, et al.	2019	OK2SHARE	Yes	The IHS-linked race code classifies individuals as AI/AN if (1) the race reported on the death certificate was AI/AN or (2) the individual record linked to IHS regardless of the original race reported on the death certificate
Duncan MH, et al.	1986	New Mexico Tumor Registry	Yes	Linked by IHS coverage
Espey DK, et al.	2008	SEER/NPCR; IHS patient registration	Yes	IHS linkage
Espey DK, et al.	2007	SEER/NPCR; CDC national vital statistics system	Yes	All records for cases diagnosed from 1995 to 2004 were linked with the IHS patient registration database to identify AI/AN cases that were misclassified as non-native.
Espey DK, et al.	2005	Death records from NCHS/SEER; IHS enrollment records	Yes	Authors limited analysis to AI/ANs who were identified on the death certificate as residing in either counties that contained federally recognized tribal lands or any adjacent county; linked to IHS
Frost F, et al.	1992	Seattle-Puget Sound Cancer Registry	Yes	IHS linkage
Gilliland FD, et al.	1998	New Mexico Tumor Registry	Yes	Multiple sources, medical records, self-report, IHS records; misclassification of AI estimated to be <1%
Gilliland FD, et al.	1998	New Mexico Tumor Registry	Yes	Multiple sources, medical records, self-report, IHS records; misclassification of AI estimated to be <1%
Gonzalez V, et al.	2022	Oklahoma Central Cancer Registry and CDC WONDER	Yes	IHS racial categories and linkage
Gopalani SV, et al.	2020	CDC Wonder/U.S. Cancer Statistics; SEER	Yes	IHS linkage
Henderson JA, et al.	2008	NPCR, SEER, and IHS records	Yes	States linked all case records with the IHS patient registration database to identify AI/AN cases misclassified as some other racial group. Analyses only included CHSDA to improve race classification
Hoffman RM, et al.	2014	NPCR and SEER/NCI; NVSS	Yes	States linked all case records with the IHS patient registration database to identify AI/AN cases misclassified as some other racial group. Analyses only included CHSDA to improve race classification
Hoopes MJ, et al.	2012	Northwest Tribal Registry; state cancer registry	Yes	IHS linkage, with NW Tribal Registry
Hoopes MJ, et al.	2010	Northwest Tribal Registry	Yes	Linked with Northwest Tribal Registry and Seattle Indian Health Board patient records
Jim MA, et al.	2014	IHS; National Vital Statistics System Mortality Files; NPCR and SEER; 43 state cancer registries	Yes	IHS linkage to NDI
Jim MA, et al.	2008	NPCR/SEER	Yes	IHS linkage to NDI
Johnson JC, et al.	2009	Michigan state cancer registry; NPCR/SEER, tribal clinic records	Yes	Registry Plus Link; linkage with tribal clinic records
Kratzer TB, et al.	2022	NPCR/SEER; linked with IHS	Yes	IHS linkage
Lee DJ, et al.	2014	Florida Cancer Data System and Florida AHCA	No	AI/AN patients identified by insurance payer information. If patients listed IHS as payer, they were included in AI/AN group
Lemrow SM, et al.	2008	NPCR or SEER; IHS patient registration database	Yes	IHS linkage
Li J, et al.	2014	Death Index, NPCR, IHS patient registration database	Yes	IHS linkage
Mahoney MC, et al.	2009	New York State Cancer Registry	No	N/A
Martinez SA, et al.	2016	OK Central Cancer Registry/OK2SHARE	Yes	IHS linkage
Melkonian SC, et al.	2022	NPCR/SEER, IHS patient registration database	Yes	IHS linkage
Melkonian SC, et al.	2020	NPCR/SEER, IHS patient registration database	Yes	IHS linkage
Melkonian SC, et al.	2021	NPCR/SEER, IHS patient registration database	Yes	IHS linkage
Norsted TL, et al.	1989	Seattle-Puget Sound Cancer Registry	Yes	Discussion section-explanation of low incidence may be due to failure to correctly report the race in hospital records
Paisano R, et al.	1997	Death certificates from CDC National Center for Health Statistics	No	Misclassification not specified addressed until conclusion but IHS and CDC worked together to analyze persons of AI/AN classification who died and were residents of counties on or adjacent to tribal reservations by the federal government (counties served by IHS)
Perdue DG, et al.	2014	NCHS/CDC; NPCR/SEER; IHS patient registration database	Yes	IHS linkage
Perdue DG, et al.	2008	NPCR/SEER; IHS patient registration database	Yes	IHS linkage
Plescia M, et al.	2014	NCHS, IHS, NDI	Yes	IHS linkage
Pope J, et al.	2021	The Military Health System Repository; defense enrollment eligibility reporting system	No	N/A
Puukla E, et al.	2004	Cancer Data Registry of Idaho, Oregon, and Washington; IHS patient registration database	Yes	Misclassification corrected through record linkages
Qian Y, et al.	2021	SEER	No	N/A
Reichman ME, et al.	2008	SEER and NPCR	Yes	IHS linkage
Samet JM, et al.	1987	New Mexico Tumor Registry; IHS hospital records	No	N/A
Schiff M, et al.	1997	New Mexico Tumor Registry; New Mexico Bureau of Vital Records and Health Statistics	No	N/A (authors state ethnic misclassification is not problematic in our incidence or mortality data)
Simianu VV, et al.	2016	Surgical Care Outcomes Assessment Program (SCOAP) in Washington State	Yes	Race and ethnicity training was provided to registrars, admitting personnel, and abstractors at each hospital. Strategies to facilitate patients’ self-report of race and ethnicity were tailored to each site. Accordingly, race and ethnicity for all patients were ascertained by using a combination of self-report, IHS insurance status, and chart abstraction.
Singh SD, et al.	2014	NCHS, SEER, NPCR, and IHS patient registration database	Yes	IHS linkage
Sugarman JR, et al.	1994	Seattle-Puget Sound Cancer Registry	Yes	In addition to studying patients initially coded as AI in the tumor registry, the authors analyzed data from persons who, although they were coded as AI according to IHS records, were coded as non-AI in the tumor registry
Sugarman JR, et al.	1996	Washington State Cancer Registry	Yes	IHS linkage
Swan J, et al.	2006	California Health Interview Survey (CHIS)	Yes	CHIS 2001 oversampled AI/AN in an effort to include a sufficient number of respondents to allow an urban–rural comparison. The sources used in this oversampling were a telephone database compiled from California Area IHS clinics, urban Indian clinics and health organizations, and membership directories of AI/AN social organizations. Used four methods for assessing AI/AN racial category
Swan J, et al.	2003	SEER/NPCR	Yes	IHS linkage
Valway S, et al.	1990	U.S. Census and NCHS	Yes	The accuracy of the reporting of race, age at death, and place of residence on state death certificates is being evaluated based on linked birth–death infant mortality data and from the 1986 National Mortality Followback Survey conducted by NCHS. The IRS funded an oversampling of AI/AN in this survey, to evaluate the accuracy of reporting of various demographic items in Indian death certificates.
Watson M, et al.	2014	NCHS/CDC, NVSC, National Death Index, IHS patient registration database	Yes	IHS linkage
Weir HK, et al.	2008	47 population-based registries, NPCR/SEER, IHS patient registration database	Yes	IHS linkage
White A, et al.	2014	NPCR/SEER; National Death Index; IHS patient registration database	Yes	IHS linkage
White MC, et al.	2014	NPCR/SEER; National Death Index; IHS patient registration database	Yes	IHS linkage
Wiggins CL, et al.	1993	New Mexico Tumor Registry	Yes	Compared cases between the Bureau of Vital Statistics and Tumor Registry database, found 95% concordance
Wiggins CL, et al.	2008	NPCR/SEER; IHS patient registration database	Yes	IHS linkage
Wiggins CL, et al.	2008	NPCR/SEER; IHS patient registration database	Yes	IHS linkage
Wilson RT, et al.	2008	NPCR/SEER; IHS patient registration database	Yes	IHS linkage
Wingo PA, et al.	2008	NPCR/SEER; IHS patient registration database	Yes	IHS linkage
Yankaskas BC, et al.	2009	North Carolina Central Cancer Registry (NCCR), Tribal Rolls	Yes	Identified counties with majority of AI belonging to the seven state-recognized, nonfederally recognized tribes; collaborated with the tribe in each county and compared incident cancer cases in NCCR to tribal rolls

AI, American Indian; AN, Alaska Native; CDC, Centers for Disease Control and Prevention; IHS, Indian Health Services; OCCR, Oklahoma Central Cancer Registry; NPCR, National Program of Cancer Registries; SEER, Surveillance, Epidemiology, and End Results; CHSDA, Contract Health Service Delivery Area; AHCA, Florida Agency for Health Care Administration; NCHS, National Center for Health Statistics; NCI, National Cancer Institute; NDI, National Death Index; NVSS, National Vital Statistics System.

For the race and ethnicity data that were collected among these articles, the most common racial group comparison, a total of 69% (38) of articles, was between AI/AN and non-Hispanic White populations.^5–7,40–44,49,51,54,56–59,62,65,66,68–72,74–76,78,80,83–85,90–94,96,97^ Three articles (5%) compared AI/AN populations with all races.^45,52,88^ Two articles (4%) compared AI/AN, non-Hispanic White (NHW), and Hispanic populations,^50,55^ and two articles (4%) compared AI/AN, non-Hispanic White, and Black populations.^95,98^ There were 10 (18%) articles that solely analyzed AI/AN populations and did not compare any other racial or ethnic group.^33,39,47,53,60,61,63,86,87,89^ The most common method of addressing racial misclassification among these articles was linkage with the IHS or tribal clinic records (45 articles or 82%), linkage with tribal registries (4 articles or 7%), or tribal rolls (1 article or 2%). Two of the remaining five articles limited their analysis to counties served by the IHS and counties adjacent to reservation or Indian lands,^45,52^ also known as Purchased/Referred Care Delivery Area (PRCDA), historically referenced as Contract Health Service Delivery Areas. PRCDA encompasses counties that contain tribal lands as well as adjacent counties to tribal lands. One article compared data between the Bureau of Vital Statistics and the New Mexico Tumor Registry and found 95% concordance.^94^ For the remaining two articles, one study limited the analysis to states with no known issues of misclassification in health records,^89^ and another study noted misclassification as a potential factor in the results of low cancer incidence, but the authors did not account for misclassification in their methods.^72^

### Articles That Did Not Discuss Misclassification

Of the 66 articles included in our analysis, we found 11 (16%) studies that did not address racial misclassification in their research (Table 1). The earliest study in this group was published in 1979,^46^ and the latest studies were published in 2021.^77,79^ The largest cohort of data among these articles was from 1955 to 2004.^67^

Six of the 11 (55%) studies did not reference racial misclassification in their studies. Among these studies, one study used IHS records to obtain data for their research. The remaining five (45%) articles used a variety of nontribal-based databases, including the National Center for Health Statistics, National Cancer Institute, state cancer registries, and Military Health System Data Repository not accounting for misclassification.

The four articles mentioned previously, which accounted for misclassification in their studies, addressed the issue in various ways. Paisano et al. noted racial misclassification as a limitation to their research, but the factor was not accounted for in the study itself.^73^ Davidson et al. did not reference racial misclassification because of how the researchers obtained their data.^48^ Data for this study were gathered from the Alaska Native Tumor Registry, which consisted of all AN patients who have been diagnosed with any cancer while being a resident in the state of Alaska. The Alaska Native Tumor Registry worked with tribal facilities in the state to ensure quality in the data. However, this linkage was not directly discussed in the study, although the report was created in partnership with the IHS. Three studies noted that racial misclassification was not an issue in their research because of how their data were obtained. Lee et al. linked the Florida Cancer Data System with the Florida Agency for Health Care Administration and identified AI/AN patients through insurance payer information.^64^ If patients indicated that the IHS was their insurance provider and that they received care from IHS facilities, they were included in the AI/AN group. The data collected in this study were not linked with IHS directly and therefore likely included a substantial level of misclassification. Both Baquet et al. and Schiff et al. noted the relative lack of racial misclassification in the New Mexico SEER Registry, because of the practice of the registry to link with IHS before publishing data, and, therefore, it was not an issue in either study.^38,82^

### CONSIDER Statement Results

Among the 66 articles, the average number of CONSIDER checklist domains was three, with a range of zero to eight domains included (Table 2). The domain most often identified was Prioritization (60), followed by Governance (47), Methodologies (31), Dissemination (27), Relationships (22), Participation (9), Capacity (9), and Analysis and Findings (8). Only two articles met the criteria for each of the eight domains.^89,98^ The articles by Creagan et al. and Qian et al. did not meet any criteria for any of the domains.^46,79^ Regarding Governance, a total of 47 of the 66 (70%) articles met the criteria.^5,6,33,37,39,41,43–45,47,49–55,57–59,61,63,67–69,73–76,78,80–83,85–98^ Articles that met the criteria for the Governance domain described agreements between research institutions and Indigenous-governing organizations for research and/or specified protection of Indigenous intellectual property and knowledge arising from the research. A total of 60 of the 66 (90%) articles met the criteria for the Prioritization domain.^5–7,33,38–45,47–63,65–72,74–78,80,82–98^ The research from those articles explicitly stated how the research aims emerged from priorities identified by Indigenous stakeholders, governing bodies, and/or empirical evidence. A total of 22 of the 66 (33%) articles met the criteria for the Relationship domain.^33,37,39,41,43,44,47,61,67,73,74,78,80,83,85,86,89,90,93,94,96–98^ These articles either worked with Tribal or Native entities for the research, or they were established members of the research team. A total of 31 of the 66 (46%) articles met the criteria for Methodologies domain.^5,33,37,39–45,47–50,52,53,61,64,67,72,74–76,78,81,85,87–91,97,98^ These articles described the rationale of methods used and implications for Indigenous stakeholders and/or incorporated impacts of colonialization, racism, and social justice as part of the discussion and results. A total of 27 of the 66 (2%) articles met the criteria for the Dissemination domain.^5,7,33,40,41,47,49,51,53,56,60,61,67–69,73–76,78,85,86,88–90,97,98^ These articles stated the relevance of the findings to relevant Indigenous governing bodies and peoples and/or implementation strategies. A total of 9 of the 66 (1.5%) articles met the criteria for the Participation domain.^33,42,47,67,83,88,89,98^ These articles discussed how data were obtained and stored. A total of 9 of the 66 (2%) articles met the criteria for the Capacity domain.^33,37,39,61,78,85,87,89,97,98^ These articles described if research teams participated in professional development to partner with Indigenous stakeholders and/or described experiences with Indigenous research capacity. A total of 8 of the 66 (1.5%) articles met the criteria for the Analysis and Findings domain.^33,67,78,83,85,88,89,98^ These articles specified how the research analysis and reporting support the research aims.

**Table 2.** CONSIDER Checklist Domain Results, *n* = 66

Authors	CONSIDER domains	Governance	Relationships	Prioritization	Methodologies	Participation	Capacity	Analysis and findings	Dissemination
Anderson RN, et al.	1					X			
Baquet CR	1			X					
Becker TM, et al	5	X	X	X	X		X		
Becker TM, et al.	3			X	X				X
Bleed DM, et al	5	X	X	X	X				X
Bliss A, et al.	3			X	X	X			
Bruegl AS, et al.	4	X	X	X	X				
Campbell JE, et al.	4	X	X	X	X				
Cobb N, et al.	3	X		X	X				
Creagan ET, et al.	0								
Creswell PD, et al.	6	X	X	X	X	X			X
Davidson AM, et al.	2			X	X				
Dougherty TM, et al.	4	X		X	X				X
Duncan MH, et al.	3	X		X	X				
Espey DK, et al.	3	X		X					X
Espey DK, et al.	4	X		X	X				X
Espey DK, et al.	3	X		X	X				
Frost F, et al.	4	X		X	X				X
Gilliland FD, et al.	2	X		X					
Gilliland FD, et al.	2	X		X					
Gonzalez V, et al.	2			X					X
Gopalani SV, et al.	2	X		X					
Henderson JA, et al.	2	X		X					
Hoffman RM, et al.	2	X		X					
Hoopes MJ, et al.	6	X	X	X	X		X		X
Hoopes MJ, et al.	2			X					X
Jim MA, et al.	2	X		X					
Jim MA, et al.	1			X					
Johnson JC, et al.	8	X	X	X	X	X	X	X	X
Kratzer TB, et al.	2			X					X
Lee DJ, et al.	1				X				
Lemrow SM, et al.	1			X					
Li J, et al.	1			X					
Mahoney MC, et al.	7	X	X	X	X	X		X	X
Martinez SA, et al.	3	X		X					X
Melkonian SC, et al.	3	X		X					X
Melkonian SC, et al.	1			X					
Melkonian SC, et al.	1			X					
Norsted TL, et al.	2			X	X				
Paisano R, et al.	3	X	X						X
Perdue DG, et al.	4	X		X	X				X
Perdue DG, et al.	5	X	X	X	X				X
Plescia M, et al.	4	X		X	X				X
Pope J, et al.	1			X					
Puukla E, et al.	7	X	X	X	X		X	X	X
Qian Y, et al.	0								
Reichman ME, et al.	3	X	X	X					
Samet JM, et al.	2	X			X				
Schiff M, et al	2	X		X					
Simianu VV, et al.	5	X	X	X		X		X	
Singh SD, et al.	1			X					
Sugarman JR, et al.	7	X	X	X	X		X	X	X
Sugarman JR, et al.	4	X	X	X					X
Swan J, et al.	6	X		X	X	X		X	X
Swan J, et al.	3	X		X			X		
Valway S, et al.	8	X	X	X	X	X	X	X	X
Watson M, et al.	5	X	X	X	X				X
Weir HK, et al.	3	X		X	X				
White A, et al.	2	X		X					
White MC, et al.	2	X		X					
Wiggins CL, et al.	2	X		X					
Wiggins CL, et al.	3	X	X	X					
Wiggins CL, et al.	3	X	X	X					
Wilson RT, et al.	3	X	X	X					
Wingo PA, et al.	6	X	X	X	X		X		X
Yankaskas BC, et al.	8	X	X	X	X	X	X	X	X

### Oral Cancer-Specific Results

Results related to oral and pharyngeal cancers were extremely limited. Among 66 articles included for final analysis for this systematic literature review, one identified oral cavity and pharynx cancer as the cancer-focus of the study.^80^ The objective of the article was to determine the incidence rates for individual anatomical sites for cancers of the oral cavity and pharynx for the six IHS regions between 1999 and 2004 for AI/AN populations.^80^ Furthermore, the authors conducted matching of NPCR and SEER data with the IHS health database to account for misclassification and analyses focused on Contract Health Service Delivery Area (CHSDA) counties.^80^ The results indicated a lower incidence rate for all cancers of the oral cavity and pharynx combined for AI/AN populations in CHSDA counties compared with non-Hispanic White populations (8.5 vs. 11.0).^80^ The authors also indicated that analyses by individual anatomical cancer sites and/or by IHS geographic regions suggest varied results.^80^

## Discussion

It is vital to ensure accurate data collection of race/ethnicity data in all health registries and databases to effectively monitor cancer trends and subsequently address cancer health disparities for minoritized populations in the United States. The gold standard for identifying cancer type is the cancer registry, which is based on medical records data.^31^ Conversely, self-reported race and ethnicity are the gold standard for capturing such information and also align with federal standards for reporting such characteristics.^31^ One study investigated the concordance between cancer registry and self-reported data regarding cancer type, race, and Hispanic ethnicity in a large, geographically diverse population from state cancer registries, including SEER and non-SEER regions in the American Cancer Society’s Studies of Cancer Survivors.^31^ Using the Study of Cancer Survivors-I (SCS-I) and SCS-II found strong concordance for White and Black survivors and weak concordance for AI/AN and Asian/Pacific Islander survivors.^31^ As members of the AI/AN populations are often misclassified in medical records by hospital personnel, it is imperative to use tools to improve accuracy such as annual linkage with the IHS. The advantage of using data linked with the IHS records is that patients receiving care from the federal agency are confirmed as AI/AN through membership of a federally recognized tribe. However, this linkage does not resolve all racial misclassification in health data. There are AI/AN patients not currently receiving or have never received care through the IHS or those who are members of a nonfederally recognized tribe that may be subject to misclassification.

In a publication by the CareQuest Institute for Oral Health in partnership with several tribal organizations, the authors provided suggestions on how to improve data quality in the collection and reporting processes.^3^ Owing to the small size of the population, many researchers misclassify AI/AN data as “other,” which obscures and skews the data regarding the entire population. To address this, the Urban Indian Health Institute suggests oversampling the AI/AN population, allowing participants to choose multiple races and ethnicities that best represent them, aggregating data over longer periods of time, and weighted sampling. Data linkages improve data quality and data integrity, but if data linkage is not available for specific health topics, then the above recommendations must be considered.

An example of real-world data linkage practices and advancing community voices and health equity can be seen at the Public Health Agencies in the state of Oklahoma, such as the Oklahoma State Department of Health and the Oklahoma Department of Mental Health and Substance Abuse Services. These state departments have been keenly aware of the issue of racial misclassification and its impact. These agencies have special tribal-liaison divisions that work closely with the SPTHB to address the issue. Great strides have been made at acknowledging the issue exists; however, much work remains in tackling sources of misclassification considering the systemic nature of the problem. It is impossible to say how many systems a record passes through before the final data point. Each one of these systems presents the opportunity for racial misclassification. Optimistically, these agencies are extremely collaborative and partner with the SPTHB in conducting regularly convening workgroups. Even specific divisions within these agencies are willing to disseminate corrected datasets. A real opportunity exists as the Oklahoma State Department of Health recognizes the issue of racial misclassification and is proactive in seeking enhanced-linked datasets. States generally wait for the CDC to conduct the linkages; however, Oklahoma proactively sends its mortality files and requests the linkages directly.^99^ Oklahoma is also the only state that has an online data query system with these linked data.^99^ Although the issue of racial misclassification is incredibly profound, Oklahoma recognizes this and is proactively seeking solutions. Through collaborations and workgroups, we have seen that as awareness spreads, professionals are open and incredibly eager for solutions.

Through partnerships with other Tribal Epidemiology Centers, the SPTHB has learned that racial misclassification is an issue they, too, are working to tackle. These Tribal Epidemiology Centers often share information and support as they work to help better their communities. The SPTHB has worked closely with the United Southern and Eastern Tribes and the National Council on Urban and Indian Health on actions for tackling racial misclassification. There has been tremendous collaboration, cooperation, and output from these partnerships. The SPTHB plans to leverage these and other partnerships to implement systematic change not just to mitigate racial misclassification once it has occurred but to reduce the incidence of it.

Nonetheless, it is important to acknowledge that the concept of race, especially for public health and medical research is, and has historically been, convoluted, complex, and at times problematic. The collection of race information for the purposes of research with the aim of categorizing various population groups has led to the marginalization of groups of people, owing to the intricacies of such categorization and historical factors. For example, the concept of race arose in the late seventeenth century, with the rise of the transatlantic slave trade, and was even used to justify slavery with groundless, racist notions such as biological inferiority.^100–102^ Furthermore, race is a social construct based on numerous secondary physical features such as skin color, and despite extensive scientific research in opposition, there is still the unsupported belief that “race,” reflects fundamental biological differences.^100,102^ In addition, and adding to the complexity, AI/AN is a political identity. In the 2020 publication of *Data and Native American Identity,* author Kimberly R. Huyser (2020) explains:

Ultimately, the American Indian and Alaska Native identity is inherently political. It is political through the formal enrollment and connection to Native Nations. For federally recognized tribes, this also confers a government-to-government relationship between the U.S. federal government and the tribal government. Thus, the complex nature of Native identification necessitates the importance of a multidimensional measurement of race— self-reported race, socially assigned race—and questions about the connection to a Native Nation or Native community. The connections to an American Indian or Alaska Native community should be tailored for each community that tap into mechanisms of mutual acknowledgment.^103^

Furthermore, in *Morton v. Mancari*, the Supreme Court established that AI/ANs can be treated differently from other U.S. citizens by the federal government even though there are antidiscrimination laws.^104,105^ The Court held that if the law or action is based on long-standing legal responsibilities toward AI/AN interests and promotes tribal self-governance, members of tribes should be considered as political groups as opposed to racial groups.^104,105^ This indicates that under the trust doctrine when members of Indian tribes receive special treatment from the federal government under the Affordable Care Act (ACA), treatment cannot be considered racial discrimination because tribes are considered political groups, not racial groups.^102^ So, the question is, if widely recognized as a political designation rather than a race category, would that change how AI/AN data are collected and shared?

Since the last treaties were signed in 1871, AI/AN populations have been burdened with inadequate access to health care resources and services.^106,107^ AI/AN health disparities in the United States are a social justice issue that should be recognized as a national priority to increase resources and services for this population. Clearly, there are educational gaps in both the fields of public health and medicine in accurately reporting self-reported race/ethnicity and accounting for misclassification in research analysis. Such topics should be at the forefront of educational curricula. Furthermore, promoting equitable research should also be at the forefront. Incorporating the CONSIDER statement was an intentional use of an Indigenous framework to strengthen research and advance Indigenous health outcomes. Future research should be led by and in partnership with Native researchers and organizations. When researchers and health clinicians are of diverse racial and ethnic backgrounds, this advances health equity work.

## Limitations

Limitations to this study include that most were retrospective observational studies, for which missing data are inevitable. Outcomes evaluated were limited to published articles and in the English language only. Regarding the CONSIDER criteria, some of the domains are difficult to establish such as Native researchers might be members of the team but not work for a tribe or tribal serving organizations.

## Conclusion

Cancer surveillance data represent real patients in the United States and should accurately reflect their identities. Adherence to self-reported race/ethnicity in data collection for research and health information is essential to understanding racial health disparities especially for many minoritized populations, such as AI/AN populations. The implications of the findings of this review are far-reaching, including how oral health data are related to cancer. Oral health is associated with cancer incidence and survival; however, misclassification adds another layer of complexity to understanding the impact of poor oral health. By using cancer data as an example, the impact of misclassification of race on AI/AN populations is evident. To ensure equitable representation of AI/AN communities, and thwart further oppression of minorities, specifically AI/AN peoples, is through accurate data collection and reporting processes. Data linkages improve data quality and data integrity.

## Authors’ Contributions

J.S., A.J.L., and J.P. conceived the study. A.J.L., A.H., and J.E.C. completed the analysis. A.J.L. led the writing.

## Ethical Approval and Consent to Participate

Ethical approval and patient consent were not required. This material is the author’s own original work, which has not been previously published elsewhere.

## Author Disclosure Statement

The authors declare no conflicts of interest.

## Funding Information

Support for this research comes from the CareQuest Institute of Oral Health through their Advancing Equity through Oral Health funding initiative.

## Abbreviations Used

AI: American Indian
AN: Alaska Native
AHCA: Florida Agency for Health Care Administration
CDC: Centers for Disease Control and Prevention
CHSDA: Contract Health Service Delivery Area
CONSIDER: CONSolIDated critERia
IHS: Indian Health Services
NCHS: National Center for Health Statistics
NCI: National Cancer Institute
NDI: National Death Index
NPCR: National Program of Cancer Registries
NVSS: National Vital Statistics System
NHW: non-Hispanic White
OCCR: Oklahoma Central Cancer Registry
PRCDA: Purchased/Referred Care Delivery Area
SEER: Surveillance, Epidemiology, and End Results
SPTHB: Southern Plains Tribal Health Board
